# Desquamated Epithelial Cells of Unstimulated Human Whole Saliva Express Both EGF Transcript and Protein

**DOI:** 10.1155/2022/3194703

**Published:** 2022-12-17

**Authors:** Alexandra Aidoukovitch, Sara Bodahl, Ellen Tufvesson, Bengt-Olof Nilsson

**Affiliations:** ^1^Department of Experimental Medical Science, Lund University, Lund, Sweden; ^2^Folktandvården Skåne, Lund, Sweden; ^3^Department of Clinical Sciences Lund, Respiratory Medicine and Allergology, Lund University, Lund, Sweden

## Abstract

**Objective:**

The aim of this study was to investigate if desquamated oral epithelial cells (DOECs) express the epidermal growth factor (EGF) and if these cells thereby may contribute to salivary EGF contents.

**Background:**

DOECs have recently been shown to harbor the antimicrobial peptide LL-37, proposing that they may also store other biologically important salivary peptides/proteins. The EGF peptide is a growth factor which plays a critical role to maintain epithelial integrity and promote epithelial healing. The EGF is produced by salivary glands, but it is not known whether DOECs contain the EGF and thereby contribute to salivary EGF levels.

**Materials and Methods:**

DOECs were isolated from unstimulated whole saliva collected from four healthy volunteers. EGF protein expression was determined in cell lysates by dot blot and ELISA. Cellular distribution of cytokeratin, the proliferation marker Ki67, and EGF immunoreactivity were assessed by immunocytochemistry. EGF gene expression was investigated by qPCR. Expression of EGF transcript and protein in DOECs was compared to that in the human cultured keratinocyte cell line (HaCaT) cells.

**Results:**

EGF protein expression was detected in DOEC cell lysates by both dot blot and ELISA. Strong cytoplasmic EGF immunoreactivity was observed in DOECs, although some cells showed only a weak immunoreactive signal for EGF. Moreover, DOECs, besides containing EGF protein, also expressed transcript for EGF. Interestingly, ELISA analysis revealed that EGF protein contents were higher in DOECs than in HaCaT cells. ELISA analysis also disclosed that EGF concentration was about 10 times higher in whole saliva compared to DOECs. EGF transcript expression was about 50% lower in HaCaT cells stimulated with high (10%) compared to low (0.1%) concentration of fetal bovine serum, representing growth-stimulated and growth-restricted conditions, respectively, implying that growth-stimulus exerts negative feedback on EGF gene activity in HaCaT cells.

**Conclusion:**

Here, we show for the first time that DOECs express the EGF, arguing that these cells contribute to salivary EGF contents and hence may play a role in gingival epithelial repair and wound healing.

## 1. Introduction

Desquamated epithelial cells and leukocytes, mainly neutrophils, constitute the cellular component of human whole saliva [1–4]. Notably, the number of leukocytes is very low in saliva from individuals with healthy gingiva, and hence, desquamated epithelial cells represent the principal cell type of whole saliva collected from healthy individuals [2]. The desquamated oral epithelial cells (DOECs) are large cells, and they show morphological signs typical for aged epithelial cells such as small nuclei and large cytoplasm [5, 6]. We have previously demonstrated that DOECs harbor the human antimicrobial peptide LL-37, suggesting that these cells also may synthesize and/or act as containers for other biologically important salivary peptides and proteins [6, 7].

The epidermal growth factor (EGF) is a peptide composed of 53 amino acids with a molecular weight of 6 kDa produced by proteolytic cleavage of a 185 kDa proform [8]. It is well recognized that the cleavage of the 185 kDa EGF proform generates many smaller products (40–100 kDa), but the further process of forming the mature, circulating 6 kDa EGF peptide is not completely understood. EGF is expressed in many different cell types, and it is found in body fluids such as plasma, urine, and saliva [9–11]. The peptide was initially isolated from tissue extracts of the mouse submandibular gland and found to promote incisor eruption and stimulate eyelid opening in new-born mice by Cohen [12]. Since then, the EGF has been demonstrated in both human submandibular and parotid gland tissues [13, 14]. There are no gender differences reported for EGF levels in human whole saliva, and moreover, no changes are observed in salivary EGF concentration with mastication [10, 14]. Interestingly, it has recently been reported that the human cultured keratinocyte cell line (HaCaT) cells express both EGF mRNA and protein and that they show a basal, constitutive release of EGF to their medium [15]. HaCaT cells are highly proliferative keratinocytes, easy to culture, and hence, they are suitable for studying gene and protein regulation in cell culture experiments.

The EGF acts as a growth factor and stimulates epithelial cell proliferation via binding and activation of its plasma membrane receptor EGFR [16, 17]. Upon ligand-binding, the EGFR is activated, and the EGF/EGFR complex possesses tyrosine kinase activity representing an early step in the downstream EGFR signaling cascade [16–19]. It has been proposed that salivary EGF maintains esophageal mucosal integrity and promotes healing and repair of the gastroduodenal mucosa, and hence, salivary EGF may exert its proproliferative effect orally but also in more distal parts of the gastrointestinal tract [10, 20, 21].

The aim of this study was to investigate the expression of EGF in DOECs isolated from unstimulated human whole saliva to assess if oral epithelial cells may contribute to salivary EGF contents. Here, we demonstrate that DOECs express both EGF mRNA and protein, and furthermore, we show that expression of the EGF transcript by human keratinocyte HaCaT cells negatively correlates with growth stimulation. Taken together, these results reveal that DOECs contribute to EGF contents of whole saliva, suggesting that DOECs may act to preserve gingival epithelial structure and function.

## 2. Materials and Methods

### 2.1. Isolation of DOECs from Whole Saliva and Culture of HaCaT Cells

Unstimulated whole saliva was collected from four systemically healthy, nonsmoking volunteers (27–60 years of age), and DOECs were isolated as described previously [6]. Neither the periodontal status of the volunteers nor their use of drugs was determined. All four individuals (three females and one male) provided informed consent, and the procedure was performed in accordance with the Swedish Ethics Review Authority directives and the World Medical Association 1964 Declaration of Helsinki. Cells were isolated on multiple occasions from each individual to maintain sufficient supply of DOECs. The volunteers spat two ml saliva into plastic tubes at about 9 o'clock in the morning, at least one hour after food intake. The samples of saliva were diluted 1 : 4 in 0.1% dithiothreitol (Sigma-Aldrich) prepared in phosphate-buffered saline (PBS, Gibco) to depolymerize mucins and reduce their viscosity to allow isolation of DOECs. Salivary samples were filtered through a nylon net filter with 60 *μ*m pore size (Merck Millipore) and then centrifuged gently at slow speed (82 *g*) at 20°C for 5 min to pellet the cells. The cell pellet was washed once with PBS and the cells counted as described in the following paragraph. For each individual, the number of cells (cells/ml) in saliva was about 400000 (donor 1), 400000 (donor 2), 50000 (donor 3), and 180000 (donor 4). Variations in salivary cell density were observed at the different times of sample collection for each individual, but the proportional relationship between individuals was consistent. DOECs were used for determination of total protein concentration, dot blot analysis, immunocytochemistry, ELISA analysis, and qPCR as described in the following paragraph.

Human keratinocyte cell line HaCaT cells were purchased from CLS Cell Lines Service GmbH and cultured in DMEM/Ham's F12 medium (1 : 1, Biowest) supplemented with 10% fetal bovine serum (FBS, Biochrom) and antibiotics (50 U/ml penicillin and 50 *μ*g/ml streptomycin, Biochrom). The cells were cultured in culture dishes (VWR) and culture well plates (Sarstedt) and kept in a water-jacketed cell incubator at 37°C under 5% CO_2_ in air. They were trypsinized (0.25% trypsin-EDTA solution, Sigma-Aldrich), counted, and reseeded upon reaching confluence and used for experiments at 60 to 80% confluence. To assess the impact of growth and proliferation on EGF expression, experiments were performed under growth-restricted (0.1% FBS) or growth-stimulated (10% FBS) conditions. HaCaT cells were used for measurement of total protein concentration, ELISA analysis, and qPCR as described in the following section.

### 2.2. Cell Counting and Determination of Total Protein Concentration

DOECs and HaCaT cells were stained with trypan blue (Sigma-Aldrich) and counted using an automated cell counter (LUNA, Logos Biosystems). For determination of total protein concentration, DOECs and HaCaT cells were washed in PBS, and HaCaT cells were scraped off the bottom of the culture well in ice-cold PBS using cell scrapers (Sarstedt) and centrifuged (82 *g*, 4°C, 5 min). DOEC and HaCaT cell lysates were prepared in ice-cold PBS by sonication (2 × 10 s), and total protein concentration in the cell lysates and in whole saliva was determined using the Bio-Rad DC protein assay kit (Bio-Rad). Each sample was analyzed in duplicate.

### 2.3. Dot Blot

DOECs were lysed in SDS sample buffer, and cell lysates were sonicated for 2 × 10 s. The cell lysates were kept on ice for 10 min and boiled for 5 min before centrifugation (16000 *g*) at 4°C for 15 min. Supernatants were collected, and total protein concentration was determined in each sample (Bio-Rad DC protein assay kit) to assure equal loading in each dot. For each dot, 1 *μ*l cell lysate was loaded onto a nitrocellulose membrane. The membranes were blocked in 0.5% casein in Tris-buffered saline (Bio-Rad) for 2 h, incubated overnight with a rabbit polyclonal EGF antibody (Novus Biologicals, catalog # NBP1-198006SS) at a dilution of 1 : 1200, and then incubated with horseradish peroxidase-conjugated anti-rabbit IgG at a dilution of 1 : 5000 for 2 h (Cell Signaling, catalog no. 7074) and West Femto chemiluminescence reagent (Thermo Fisher Scientific). EGF immunoreactivity was visualized using a LI-COR Odyssey FC instrument (LI-COR Biosciences).

### 2.4. Immunocytochemistry

DOECs were dispersed in PBS, counted, and transferred to microscope slides using cytospin centrifugation at 450 *g* for 6 min (5000 cells per spot). HaCaT cells were cultured on coverslips. The cells were fixed in 4% paraformaldehyde (Polysciences) for 10 min, permeabilized with 0.2% Triton X-100 (Sigma-Aldrich) for 10 min, and then incubated with 2% bovine serum albumin (Sigma-Aldrich) for 2 h to block unspecific binding sites. Cells were incubated overnight at 4°C with the rabbit polyclonal EGF antibody (Novus Biologicals, catalog no. NBP1-198006) at a dilution of 1:100, rabbit polyclonal Ki67 antibody (Abcam, catalog no. ab15580) at a dilution of 1:500, and mouse monoclonal cytokeratin antibody (Dako, catalog no. M082101-2) at a dilution of 1:400, followed by incubation for 1 h with anti-rabbit or anti-mouse IgG conjugated with ALexa Fluor 488 (Thermo Fisher Scientific) for detection of the immunoreactive signal. Coverslips were then mounted on microscope slides using mounting medium which contains the nuclear marker DAPI (Fluoroshield, Sigma-Aldrich). Immunoreactivity and DAPI staining were assessed using a fluorescence microscope with appropriate filter settings (Olympus BX60). For negative controls, the primary antibodies were omitted. No immunoreactivity was observed after omission of the primary antibody.

### 2.5. ELISA

DOEC and HaCaT cell pellets were dispersed in ice-cold PBS. The cells were counted using the automatic Luna cell counter and thereafter sonicated (2 × 10 s). Cell lysates were centrifuged (1310 *g*, 4°C, 5 min), and supernatants were collected for ELISA analysis. ELISA analysis was also performed in unstimulated whole saliva. ELISA was performed using the Quantikine ELISA human EGF immunoassay as recommended by the manufacturer (R&D Systems, catalog number DEG00). Data was normalized both to the number of cells and total protein concentrations (Bio-Rad DC protein assay kit). Each sample was analyzed in duplicate.

### 2.6. Quantitative Real-Time RT-PCR

DOECs and HaCaT cells were lysed in QIAzol lysis reagent and RLT lysis buffer, respectively (both from Qiagen), and RNA was extracted and purified using RNeasy kits and the QIAcube platform according to instructions by the manufacturer (Qiagen). DNase 1 was present during extraction and purification of RNA according to instructions (Qiagen). RNA concentration and quality were investigated by using a NanoDrop 2000C spectrophotometer (Thermo Fisher Scientific). QIAzol has successfully been used as cell lysis reagent for extraction and purification of RNA from cell pellets of human whole saliva [22]. Gene expression was determined with one-step qPCR in a StepOne Plus real-time thermal cycler (Applied Biosystems) using QuantiFast SYBR Green RT-PCR Kit (Qiagen) and QuantiTect primer assays, and gene expression was calculated applying the delta-delta cycle threshold (Ct) method with glyceraldehyde 3-phosphate dehydrogenase (GAPDH) or 18S ribosomal RNA (18S) as reference genes according to Pfaffl [23]. Each sample was analyzed in duplicate. Primers for EGF (Hs_EGF_1_SG), GAPDH (Hs_GAPDH_2_SG), and 18S (Hs_RRN18S_1_SG) were purchased from Qiagen. No or insufficient amplification was observed in negative controls where RNA was replaced with RNase-free water (Qiagen). The Ct values for all targets in RNase-free water and RNA samples in both DOECs and HaCaT cells are presented in Table 1.

### 2.7. Statistics

Summarized data are presented as means ± SEM. Statistical significance was calculated by Student's two-tailed *t* test for single comparisons between the two groups (GraphPad Prism9 and GraphPad Software). *P* values less than 0.05 were regarded to denote statistical significance.

## 3. Results

### 3.1. Dot Blot Discloses EGF Immunoreactivity in DOEC Cell Lysates

**Figure 1:**
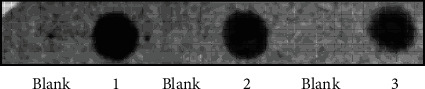
EGF immunoreactivity is detected in cell lysates of DOECs by using dot blot. Cells were isolated from human unstimulated whole saliva, lysed in SDS sample buffer, sonicated, boiled, and then the cell lysates were centrifuged, and the supernatants were collected for analysis. EGF immunoreactivity was assessed in samples prepared from three of the four individuals (1, 2, and 3) included in the study. Each sample was analyzed in duplicate. SDS sample buffer was utilized as blank.

In the first experiments, we prepared cell lysates of DOECs collected from three of the four individuals included in the study and assessed immunoreactivity in these lysates using dot blot. For dot blot analysis, EGF immunoreactivity was observed in cells obtained from all three individuals, whereas blanks were negative as predicted (Figure 1).

### 3.2. DOECs Express Cytoplasmic Immunoreactivity for the EGF

**Figure 2:**
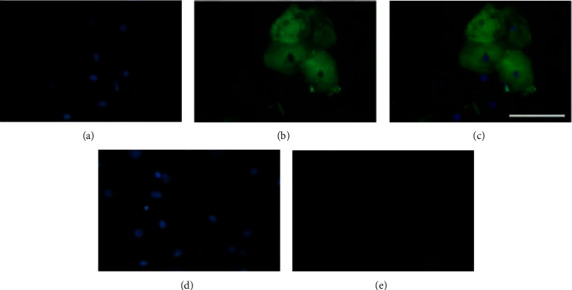
Immunocytochemistry reveals EGF immunoreactivity in DOECs. (a–e) Cells were transferred to microscope slides using the cytospin technique and fixed. The same cells were double stained for nuclei using the nuclear marker DAPI (a, d) and for EGF immunoreactivity (b, e). Overlay of DAPI stain and EGF immunoreactivity is shown in panel (c). For negative controls, the primary EGF antibody was omitted (d, e). The bar in panel (c) represents 100 *μ*m for all panels. Each sample was analyzed in duplicate or triplicate. EGF immunoreactivity was assessed in cells isolated from one of the four volunteers included in the study. Pictures were obtained using an Olympus BX60 fluorescence microscope equipped with a digital camera and appropriate filter settings.

**Figure 3:**
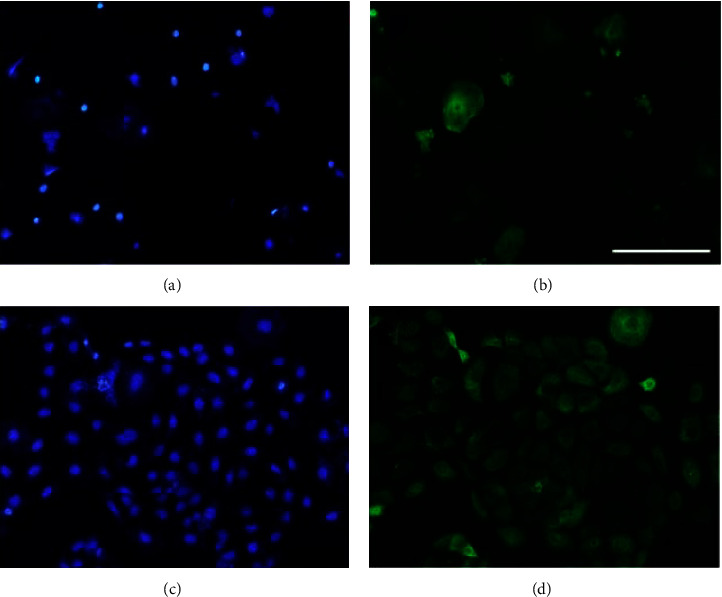
Both DOECs and HaCaT cells express immunoreactivity for the keratinocyte marker cytokeratin. (a–d) DOECs (a, b) were transferred to microscope slides by cytospin, and HaCaT cells (c, d) were cultured on coverslips. Cells were fixed and double stained for the nuclear marker DAPI (a, c) and for cytokeratin immunoreactivity (b, d). The bar in panel (b) represents 150 *μ*m for all panels. Each sample was analyzed in duplicate.

In the next experiments, we assessed EGF expression in DOECs by immunocytochemistry. The same cells were double stained for nuclei using the nuclear marker DAPI and for EGF immunoreactivity. As seen in Figure 2, DOECs are big cells with a small nucleus and extensive cytoplasm as described before [6]. Strong immunoreactivity for EGF was detected in the cytoplasm of many cells, whereas some cells showed a weak cytoplasmic signal (Figures 2(a)–2(c)). The immunoreactive signal for EGF was distributed throughout the whole cytoplasm of the EGF positive cells (Figures 2(a)–2(c)). Very weak or no EGF immunoreactivity was observed in the nuclei (Figures 2(a)–2(c)). No immunoreactivity for EGF was observed following omission of the primary EGF antibody (Figures 2(d) and 2(e)). DOECs consist of oral keratinocytes, and in the following experiments, we compared EGF protein and gene expression in DOECs with that in the human keratinocyte cell line HaCaT cells. Firstly, we assessed expression of the keratinocyte marker cytokeratin in DOECs and HaCaT cells for characterization. Cytoplasmic immunoreactivity for cytokeratin was observed both in DOECs and HaCaT cells, but some DOECs only showed weak staining (Figures 3(a)–3(d)).

### 3.3. ELISA Analysis Shows that DOECs Contain EGF Protein

**Figure 4:**
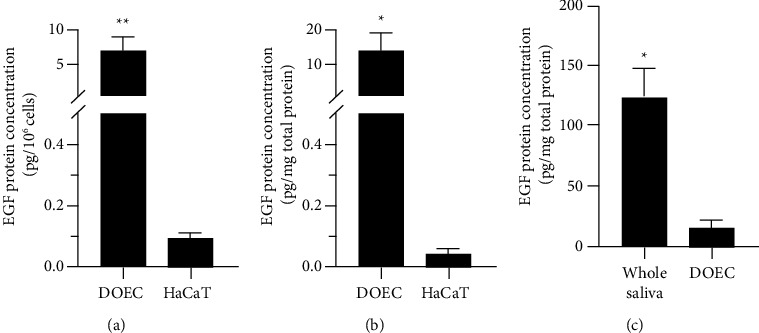
DOECs contain EGF protein as shown by ELISA analysis. (a, b) EGF protein concentration was investigated in DOECs and human keratinocyte HaCaT cell lysates with ELISA and expressed as pg/10^6^ cells (a) or pg/mg total protein (b). (c) EGF concentration (pg/mg total protein) determined in unstimulated whole saliva and DOECs using ELISA. Each sample was analyzed in duplicate. Values are presented as means ± SEM for 4 (number of individuals) or 6 (number of wells with HaCaT cells) observations in each group. Statistical significance was calculated by Student's two-tailed *t* test for single comparisons between the two groups (DOECs *vs*. HaCaT cells and whole saliva *vs*. DOECs) as appropriate. ^*∗*^ represents *P* < 0.05, and ^*∗∗*^ represents *P* < 0.01.

Next, we performed the ELISA for the EGF in cell lysates of DOECs and compared their levels of the EGF with those in HaCaT cells. EGF levels in the cell lysates were normalized both to the number of cells (Figure 4(a)) and total protein concentration (Figure 4(b)). ELISA analysis showed that the DOECs isolated from all four volunteers included in the study contained the EGF protein (Figures 4(a) and 4(b)). Interestingly, EGF contents were several folds higher in DOECs compared to HaCaT cells, both expressed per cell (Figure 4(a)) and per mg total protein (Figure 4(b)). In addition, we determined EGF concentration in unstimulated whole saliva for comparison. As seen in Figure 4(c), EGF concentration, normalized to total protein concentration, was about 10 times higher in whole saliva compared to DOECs.

### 3.4. DOECs Express Transcript for the EGF

**Figure 5:**
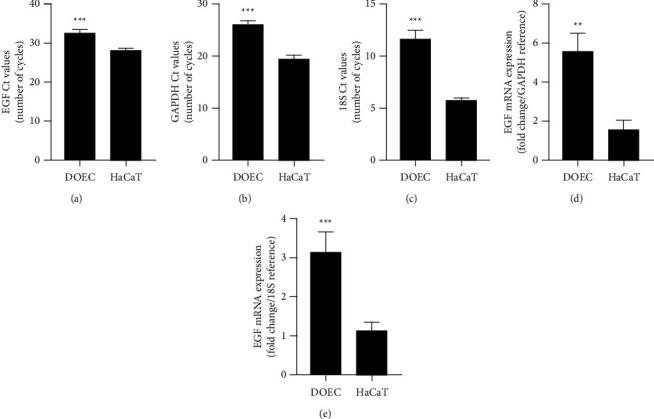
DOECs express the EGF gene as shown by qPCR analysis. (a–e) EGF transcript is detected in DOECs isolated from all 4 volunteers included in the study by qPCR analysis. DOECs have higher Ct values for the EGF (a), the GAPDH (b), and the 18S (c) genes compared to HaCaT cells. Note that a high Ct value represents low gene activity and *vice versa*. Calculation of EGF gene expression by the delta-delta Ct method and the Pfaffl equation with, respectively, GAPDH and 18S as reference genes shows that relative EGF gene expression is higher in DOECs than in HaCaT cells (d, e). Each sample was analyzed in duplicate. DOECs were isolated from all 4 individuals included in the study. Values are presented as means ± SEM for three experiments. Statistical significance was calculated by Student's two-tailed *t* test for single comparisons between the two groups (DOECs *vs*. HaCaT cells) as appropriate. ^*∗∗*^represents *P* < 0.01, and ^*∗∗∗*^represents *P* < 0.001.

In the next experiments, we assessed mRNA levels for the EGF in DOECs and compared their EGF gene expression with that in HaCaT cells using the Ct values obtained in the qPCR analysis. Low and high Ct values for the target gene represent high and low gene expression, respectively. Importantly, mRNA for the EGF gene was detected in DOECs isolated from each of the four volunteers included in the study (Figures 5(a), 5(d), and 5(e)). Notably, the Ct values for the EGF gene were higher in DOECs compared to HaCaT cells (Figure 5(a)). DOECs also had higher Ct values than HaCaT cells both for the glycolysis associated GAPDH and the ribosomal 18S reference genes, implying reduced metabolism and cell viability in DOECs (Figures 5(b) and 5(c)). Analysis of EGF gene expression, applying the delta-delta Ct method and the Pfaffl equation [23], showed that mRNA levels for EGF, normalized to either GAPDH or 18S as reference genes, were higher in DOECs compared to HaCaT cells (Figures 5(d) and 5(e)).

### 3.5. EGF Gene Expression Is Lower in Growth-Stimulated than in Growth-Restricted HaCaT Cells

**Figure 6:**
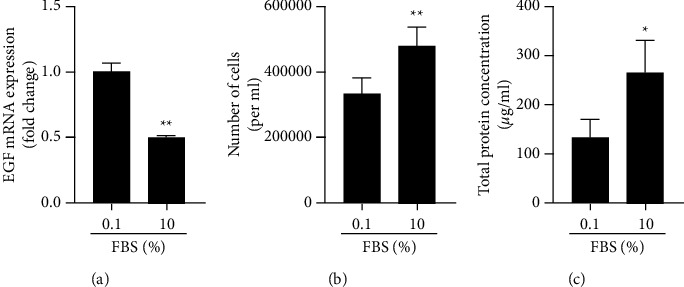
EGF gene expression is lower in growth-stimulated than in growth-restricted HaCaT cells. (a–c) Cells were incubated in the FBS-free culture medium for 8 h to standardize the experimental conditions and then stimulated with either low (0.1%) or high (10%) concentration of FBS for 24 h representing growth-restricted and growth-stimulated conditions, respectively. EGF mRNA levels were determined by qPCR using the delta-delta Ct method (a). Cells were stained with trypan blue, and the number of cells was counted using an automatic cell counter (b). Total protein concentration in cell lysates was investigated using a Bio-Rad DC protein assay kit (c). Experiments were performed three times in duplicate or triplicate. Values are presented as means ± SEM for three experiments in each group. Statistical significance was calculated using Student's two-tailed *t* test for single comparison between the two groups (0.1 *vs*. 10% FBS) as appropriate. ^*∗*^represents *P* < 0.05, and ^*∗∗*^represents *P* < 0.01.

**Figure 7:**
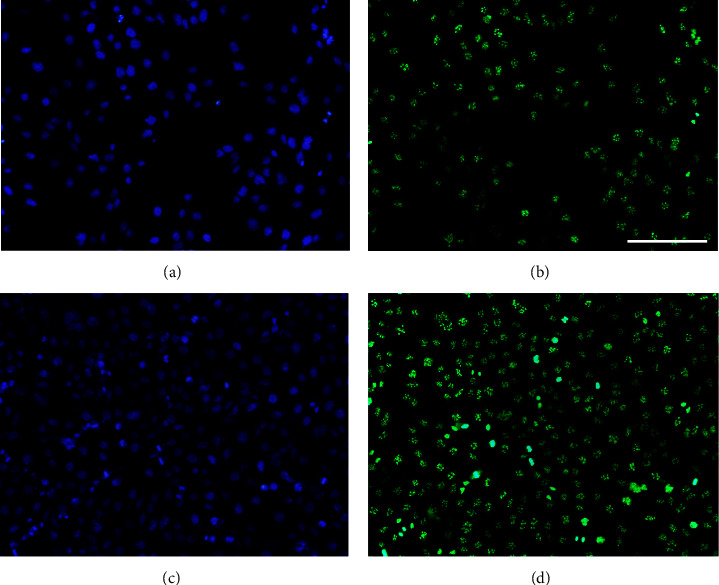
Immunocytochemistry discloses intense nuclear staining for the proliferation marker Ki67 in HaCaT cells stimulated with 10% FBS. (a–d) Cells were incubated in the FBS-free culture medium for 8 h to standardize the experimental conditions and then stimulated with either low (0.1%, (a, b)) or high (10%, (c, d)) concentration of FBS for 24 h HaCaT cells were cultured on coverslips and fixed. The same cells were double stained for nuclei using the nuclear marker DAPI (a, c) and for Ki67 immunoreactivity (b, d). Bar in panel (b) represents 150 *μ*m for all panels. Each sample was analyzed in duplicate.

Since EGF regulates epithelial cell proliferation and overall gene expression is low in DOECs compared to HaCaT cells, we chose to assess the impact of growth stimulation on EGF gene expression in HaCaT cells. In these experiments, the cells were preincubated in the FBS-free culture medium for 8 h to standardize the experimental conditions, before they were incubated for 24 h in the medium with either low (0.1%) or high (10%) concentration of FBS, representing growth-restricted and growth-stimulated conditions, respectively. Interestingly, EGF gene expression negatively correlated with growth stimulation, i.e., FBS concentration (Figure 6(a)). EGF mRNA levels were about 50% lower in cells stimulated with 10% FBS than in cells incubated with 0.1% FBS (Figure 6(a)). Next, we assessed cell number and total protein concentration in cell lysates of HaCaT cells to confirm the FBS-induced growth stimulation. Stimulation with 10% FBS enhanced the cell number by about 50% and total protein concentration by about 2 times compared to 0.1% FBS, demonstrating that 10% FBS triggers proliferation in HaCaT cells (Figures 6(b) and 6(c)). Finally, we performed immunostaining with the proliferation marker Ki67 to assess HaCaT cell proliferation in response to stimulation with either 0.1 or 10% FBS for 24 h. Nuclear staining for Ki67 was observed in cells stimulated with both 0.1 and 10% FBS (Figures 7(a)–7(d)), but the immunoreactive signal for Ki67 was more intense in cells stimulated with 10% FBS compared to those stimulated with 0.1% FBS (Figures 7(a)–7(d)).

## 4. Discussion

In the present study, we show for the first time that DOECs contain EGF, suggesting that they can contribute to the EGF contents of whole saliva. We demonstrate, using dot blot, immunocytochemistry, and ELISA, that DOECs contain EGF protein, and moreover, we also show that DOECs express mRNA for EGF, and thus, they may possess EGF gene activity. Notably, EGF immunoreactivity was displayed in DOECs using two different primary EGF antibodies, one for dot blot and immunocytochemistry and another for ELISA analysis, and hence, EGF protein is detected in DOECs by utilizing many different techniques and more than one primary EGF antibody, strengthening our observations. As mentioned before, we use different methods (dot blot, immunocytochemistry, and ELISA) to detect the EGF in DOECs. Interestingly, we observe similar EGF expression in DOECs isolated from the different individuals included in the study using these techniques. Immunocytochemistry allows us to assess cellular localization of the EGF, whereas dot blot and ELISA are sensitive methods to determine EGF immunoreactivity and absolute concentrations of the EGF. In the present study, western blot was not used for assessment of EGF expression. This method has the advantage that it separates proteins/peptides by charge and size, and thus, it permits identification of immunoreactivity of the protein/peptide of interest with high specificity. However, since EGF is produced as a 185 kDa proform which is processed to the biologically active 6 kDa EGF peptide, western blot produces many EGF immunoreactive bands, and thus, it is very difficult to conclude which is/are the relevant and important one(s), representing a major disadvantage.

Importantly, EGF expression in DOECs is demonstrated on both protein and mRNA levels. Therefore, it is reasonable to propose that keratinocytes produce the EGF, at least when they are in their natural context within the oral mucosa. Importantly, ELISA analysis disclosed that DOECs contained more EGF protein compared to the human keratinocyte cell line HaCaT cells, both expressed per cell and per mg total protein, indicating that DOECs may bind and/or internalize exogenous salivary EGF, probably produced by the salivary glands, after desquamation when they are present in saliva. Furthermore, we show that EGF concentration is much higher in whole saliva compared to DOECs, implying that although DOECs may contribute to total salivary EGF, salivary EGF mainly originates from other sources than DOECs.

Interestingly, some DOECs show strong immunoreactive staining for the EGF in their cytoplasm, whereas others possess only a weak EGF signal, suggesting that there are two different populations of DOECs present in human saliva, represented by cells which are rich in EGF and those which contain low amounts of the peptide. Importantly, EGF immunoreactivity in DOECs is distributed in the whole cytoplasm and not concentrated to the plasma membrane and the outer surface of the cell, arguing that the EGF is not binding to the cell surface, but it is preferably produced and/or internalized by the cells. Notably, we can draw this conclusion since the cells are permeabilized when prepared for immunocytochemistry. Moreover, we believe it is reasonable to propose that DOECs spontaneously release the peptide, although DOECs also probably contribute to salivary contents of the EGF via cell lysis.

It has recently been reported that HaCaT cells express EGF transcript and protein, and in the present study, we confirm these findings [15]. HaCaT cells have been widely used to study functional properties of oral keratinocytes in both health and disease, and they are considered representative for human keratinocytes *in situ* [24–27]. Here, we demonstrate that EGF gene expression is higher in growth-restricted than in growth-stimulated HaCaT cells, implying that transcriptional activity of the EGF gene negatively correlates with growth stimulation. Hence, the low EGF gene activity that we observe in growth-stimulated cells probably reflects a negative feed-back mechanism which may function to reduce EGF production and thus keratinocyte proliferation, through a paracrine mechanism.

For the gingival epithelium, DOECs may harbor and transfer EGF to damaged regions of the epithelium, where the peptide can be released and contribute to maintenance of epithelial cell integrity and also enhance epithelial healing [28]. Interestingly, the periodontitis-associated pathogen *P*. *gingivalis* has been shown to inhibit EGF-induced epithelial cell proliferation and migration, offering a potential mechanism that can be responsible for the reduced gingival healing and tissue damage in periodontitis [29]. In this context, it is notable that the periodontal status of the volunteers included in this study is not determined, representing a limitation. Furthermore, we do not assess if they use drugs, denoting another limitation of this study. Noteworthy, drugs can affect both the volume of saliva and its quality and probably also the number of DOECs. Since the EGF stimulates epithelial cell proliferation, it may promote oral epithelial repair and improve oral health via this mechanism. Hence, supplementation with exogenous EGF may have a beneficial effect on oral health, although there is a risk for unwanted EGF-induced stimulation of epithelial cell proliferation. Interestingly, reduction of salivary EGF levels in Sjögren's syndrome patients seems to correlate with lowered oral health-associated quality of life [30].

In summary, we conclude that DOECs harbor the EGF, and we propose that these cells contribute to salivary EGF contents and hence play a role in maintaining gingival epithelial integrity and/or stimulating gingival epithelial repair processes.

## Figures and Tables

**Table 1:** Ct values for the epidermal growth factor (EGF), glyceraldehyde 3-phosphate dehydrogenase (GAPDH), 18S ribosomal RNA (18S) in negative controls (RNAse-free water), and RNA samples for DOECs and HaCaT cells. Gene expression was assessed in DOECs isolated from all 4 individuals included in the study. Values are presented as means ± SEM for three experiments. ND: not detected.

Sample	Ct value EGF	Ct value GAPDH	Ct value 18S
DOEC	32.70 ± 0.84	26.20 ± 0.59	11.69 ± 0.81
HaCaT	28.85 ± 0.30	20.76 ± 0.49	5.57 ± 0.18
Negative control	ND	ND	27.92 ± 0.11

## Data Availability

The data that support the findings of this study are available from the corresponding author upon reasonable request.
